# *Drosophila melanogaster* experimental model to test new antimicrobials: a methodological approach

**DOI:** 10.3389/fmicb.2024.1478263

**Published:** 2024-11-06

**Authors:** Maria Vidal, Marta Arch, Esther Fuentes, Pere-Joan Cardona

**Affiliations:** ^1^Microbiology Department, Laboratori Clínic Metropolitana Nord, Germans Trias i Pujol University Hospital (HUGTP), Badalona, Catalonia, Spain; ^2^Department of Genetics and Microbiology, Universitat Autònoma de Barcelona, Bellaterra, Catalonia, Spain; ^3^Tuberculosis Research Unit, Germans Trias i Pujol Research Institute (IGTP), Badalona, Catalonia, Spain; ^4^Comparative Medicine and Bioimage Centre of Catalonia (CMCiB), Germans Trias i Pujol Research Institute (IGTP), Badalona, Catalonia, Spain; ^5^Centro de Investigación Biomédica en Red en Enfermedades Respiratorias (CIBERES), Instituto de Salud Carlos III (ISCIII), Madrid, Spain

**Keywords:** *Drosophila melanogaster*, antimicrobials, resistance, screening, 3R policy, innate immunity, host-directed therapies, preclinical

## Abstract

Given the increasing concern about antimicrobial resistance among the microorganisms that cause infections in our society, there is an urgent need for new drug discovery. Currently, this process involves testing many low-quality compounds, resulting from the *in vivo* testing, on mammal models, which not only wastes time, resources, and money, but also raises ethical questions. In this review, we have discussed the potential of *D. melanogaster* as an intermediary experimental model in this drug discovery timeline. We have tackled the topic from a methodological perspective, providing recommendations regarding the range of drug concentrations to test based on the mechanism of action of each compound; how to treat *D. melanogaster*, how to monitor that treatment, and what parameters we should consider when designing a drug screening protocol to maximize the study’s benefits. We also discuss the necessary improvements needed to establish the *D. melanogaster* model of infection as a standard technique in the drug screening process. Overall, *D. melanogaster* has been demonstrated to be a manageable model for studying broad-spectrum infection treatment. It allows us to obtain valuable information in a cost-effective manner, which can improve the drug screening process and provide insights into our current major concern. This approach is also in line with the 3R policy in biomedical research, in particular on the replacement and reduce the use of vertebrates in preclinical development.

## 1 Introduction

Nowadays, our society is threatened by antimicrobial-resistant (AMR) infections (Global Antimicrobial Resistance and Use Surveillance System (GLASS) Report 2022, 2022). A systematic review written by Antimicrobial Resistance Collaborators in 2022 remarked an estimation of 1.27 million deaths attributable to bacterial AMR in 2019 (Murray et al., 2022). Experts estimate that, by 2050, 10 million people could die annually from AMR (O’Neill, 2016). This makes antibiotic resistance an urgent global public health problem and claims the urgency to search for new effective drugs against the multiple infectious agents that surround us and cause disease. New treatment research is based on drug-toxicity assays, efficacy and efficiency studies, description of the mechanism of action of the compounds, and pharmacokinetic and pharmacodynamic studies. Nevertheless, traditionally new compound screening methodology is going from *in vitro* with cell culture systems to preclinical assessment in experimental mammal models. While the first ones do not capture the complexity of an infected host, mammal models are strongly ethically regulated, apart from being costly and time-consuming, so they do not allow high-throughput screening of compounds and make it difficult to implement sex equality in the experiments without dramatically increase the cost (Xiu et al., 2022).

The 3Rs strategy (Replacement, Reduction, and Refinement), first promoted by William Russel and Rex Burch in 1959, has as its main objective to improve the welfare of animals used in research (Hubrecht and Carter, 2019). The use of some animals that, based on current scientific thinking, are not considered capable of experiencing suffering are encompassed in the replacement strategy, such as Drosophila, nematode worms, and social amoebae, as well as methods that avoid the use of animals such as organoids and mathematical and computer models.^1^ In this review, we will be talking about *Drosophila melanogaster* as good model to be used in AMR research as a complement and partial replacement of mammal models in several points of the drug discovery research chronogram. Extended reviews have been done about this experimental model addressing its features and its usefulness in different research fields (Arch et al., 2022; Xiu et al., 2022). In literature, their use as models of human diseases and opportunities for therapeutic discovery has been discussed for central nervous system disorders, inflammatory disorders, cardiovascular disease, cancer or diabetes (Pandey and Nichols, 2011; Munnik et al., 2022). Nevertheless, the use of the *D. melanogaster* model as a complement for *in vitro* studies in high throughput screening of drugs tackling microbial infections needs further exploration. In this article, we discussed the advantages of the fly model to work as a screening platform in drug discovery against infections. We dissected what makes it a good model for the study of host-pathogens interactions and its treatment, as well as all the previous cases present in the literature that justify it. As a novelty, this review was done from a methodological point of view to understand which is the better option to perform a compound screening for curing infections in a cost-efficient way.

## 2 *Drosophila melanogaster* as a good model for the screening of drugs against infections

*Drosophila melanogaster* is a powerful model, traditionally used in genetics, evolution, and developmental biology, with notable characteristics compared to other animal models (Xiu et al., 2022). The physiological characteristics that justify the fruit fly as a good experimental model are its short life-cycle, small size, and its larger number of offspring; the low cost of the laboratory maintenance and rear of the animal model; the fact that *D. melanogaster* has a properly characterized genome and conserved genes with mammals that allows easily genetic manipulation and that they have simpler safety consideration and ethical related concerns as compared to vertebrate model organisms (Tzelepis et al., 2013; Younes et al., 2020).

Indeed, *D. melanogaster* has functional homologs for 75% of disease-causing genes in humans and a high degree of evolutionary conservation with mammals not only in many components of the innate immune system such as immune cascades, signal transduction pathways, and transcriptional regulators; but also, in tissues such as the gut, which digests and absorbs nutrients and has a well-controlled regulation system (Lee and Min, 2019). In addition, *D. melanogaster* can host a large diversity of infectious agents either Gram-positive or Gram-negative bacteria, fungi, and viruses (Younes et al., 2020). Several infection models have been already established in *D. melanogaster* such as the infection with *Mycobacterium marinum*, *Mycobacterium abscessus*, *Candida albicans,* and *Staphylococcus aureus*, among others (Dionne et al., 2003; Needham et al., 2004; Glittenberg et al., 2011; Oh et al., 2013b). All of this together make the fruit fly a good model for studying innate immune responses and host-pathogen interactions.

*Drosophila melanogaster* has a complete and conserved innate immune system. As the elucidation of all molecular mechanisms behind immune response in *D. melanogaster* is not the main objective in this review, we leave here some other extended reviews of interest that perfectly explain everything about the topic (Buchon et al., 2014; Arch et al., 2022; Xiu et al., 2022). Briefly, *D. melanogaster* lacks an adaptive immune response, and its immune interactions rely exclusively on innate immunity with both the humoral and cell-mediated host defence factors against intracellular and extracellular external agents. As in vertebrates, *D. melanogaster* innate immune system is also divided into humoral and cellular responses. On one hand, the humoral innate immune response in *Drosophila* is mainly mediated by the production of Antimicrobial Peptides (AMPs). These, are produced as a result of the expression of pathways related to immunity in flies that are conserved in humans such as Toll, the homolog of the mammal Toll-like/Interleukine-1 receptor signaling pathway; IMD pathway, the homolog of the tumor necrosis factor receptor 1 signaling pathway; and a simpler but complete JAK/STAT signaling pathway (Buchon et al., 2014). On the other hand, the cellular innate response consists of phagocytosis primarily conducted by the macrophage-like immune cells, the plasmatocytes. This is complemented by other defence mechanisms such as encapsulation, conducted by lamellocytes (similar to a mammal natural killer cell) and plasmatocytes; and melanisation carried out by crystal cells, the homolog of a granulocyte. Also, when suffering a viral infection *D. melanogaster* activates interference RNA production (Younes et al., 2020).

Traditional high-throughput screening for small molecules in therapeutic discovery often results in ineffective or toxic compounds when tested in whole-animal models, wasting money and resources on drugs that lack therapeutic relevance (Munnik et al., 2022). This highlights the need to reconsider preclinical screening practices. With all of the above, *D. melanogaster* is a good model to enhance the discovery of higher-quality drug leads and improve the translation of preclinical findings into clinical efficacy, reducing wasted resources in drug development. Flies can be administered drugs in a variety of ways and then be phenotypically and physiologically monitored for treatment efficacy or toxicity. Several other reviews support the use of the Drosophila model as a screening platform in drug discovery (Pandey and Nichols, 2011; Tzelepis et al., 2013; Su, 2019; Munnik et al., 2022; Xiu et al., 2022).

### 2.1 *Drosophila melanogaster* compared with other cost-effective animal models: *Caenorhabditis elegans* and *Galleria mellonella*

With all of the above, *D. melanogaster* emerges as a promising model for discovering higher-quality drug leads. However, *Caenorhabditis elegans* and *Galleria mellonella* also address time and cost concerns associated with mammal models following the principle of the 3Rs. These models have played a crucial role in improving the understanding of host-pathogen interactions and innate immune responses, and their roles in drug screening have been extensively discussed (Giacomotto and Ségalat, 2010; Pandey and Nichols, 2011; Pukkila-Worley and Ausubel, 2012; Tsai et al., 2016; Tran and Luallen, 2024). Therefore, it is important to consider the advantages and disadvantages of *D. melanogaster* compared with these established models when screening drugs against infectious diseases.

On the one hand, *C. elegans* is suitable for high-throughput screening, given its small size, rapid life cycle, and transparency, which allow for automated dispensing, image analysis, and efficient screening of thousands of compounds in 348-well plates (Giacomotto and Ségalat, 2010). It shares approximately 65% homology with human disease-causing genes; however, it might be difficult to predict mammalian bioactivity given the anatomical simplicity of *C. elegans* relative to mammals. In addition, its thick cuticle hinders compound absorption, and its small size complicates measuring concentrations (Pukkila-Worley and Ausubel, 2012). In this regard, the fly digestive tract is closer to mammals’ one, and treatment efficacy assays in this model also confer information about the PK-PD, gut permeability, location, and biodisponibility of the drug in a specific place in the host, mimicking closely what happens in mammals (Lee and Min, 2019). Additionally, flies drive sex-specific responses while *C. elegans* do not have a male/female sexual differentiation. This could be advantageous for simplicity, but it could also be a disadvantage when assessing possible sex-dependent treatment features such as dose, concentration, or time of exposure (Pandey and Nichols, 2011; Arch et al., 2023). Regarding innate immunity, *C. elegans* lacks key components of the mammalian immune system, such as vertebrate cytokines, cellular immunity, inflammasome, complement, and melanization pathways which *D. melanogaster* and *G. mellonella* have (Giammarino et al., 2024). It also lacks essential pattern recognition receptors (PRRs) such as Toll-like receptors (TLRs), and transcription factors like NF-κB and MYD88 (Tran and Luallen, 2024). Instead, *C. elegans* relies on GATA transcription factors (e.g., ELT-2) and evolutionarily conserved pathways such as p38 MAPK, *β*-catenin, and FOXO to regulate immune responses. The TFEB/HLH-30 transcription factor governs autophagy (Pukkila-Worley and Ausubel, 2012). Overall, the simplicity and ease of handling of *C. elegans* make it an excellent tool for successfully screening thousands of compounds.

On the other hand, the larval stage of *Galleria mellonella* is also suitable for biochemical analyses, pathogen interaction studies, and drug screening. This model is easier to maintain and handle compared with *D. melanogaster*. In addition, one of *G. mellonella*’s key advantages is its ability to survive at 37°C, which mirrors the optimal temperature for many human pathogens (Tsai et al., 2016). Yet, unlike *D. melanogaster*, which can survive a large inoculum of the pathogen, *G. mellonella* is highly susceptible to low doses of infection by numerous pathogens (Glavis-Bloom et al., 2012). *G. mellonella* larvae infection model is usually experimentally used at the final instar stage, which lasts approximately 8.40 days, limiting the evaluation of chronic infections and treatments that require longer exposure times (Serrano et al., 2023; Wojda et al., 2020). In this model, the most common route of drug administration is through systemic injection, which provides precise control of the dosage given to each individual but lowers the throughput (Tsai et al., 2016). This differs from the *D. melanogaster* and *C. elegans* models, where oral administration is more commonly used (Glavis-Bloom et al., 2012; Tzelepis et al., 2013). Immune responses in *G. mellonella* share key components with the mammalian innate immune system, such as cellular responses driven by hemocytes involved in processes like phagocytosis, nodulation, and encapsulation (Tsai et al., 2016); and humoral responses triggered when pathogens are recognized by pathogen recognition receptors (PRRs), such as Toll-like receptors, leading to the release of antimicrobial peptides (Asai et al., 2023). However, *G. mellonella* lacks the resources found in other models, such as stock centers, standardized procedures, transgenic strains, and full genome sequencing, making it less versatile for certain types of research (Giammarino et al., 2024). Despite all this, *G. mellonella* has made a space for itself as a potential model to be used in the drug screening timeline.

All in all, the selection of which model organism to use depends on the nature of the disease being studied, the scientific questions being asked, and the type of screening procedure desired. In general terms, *C. elegans* and *G. mellonella* are frequently used for early toxicity screening and have become viable high-throughput options for evaluating drug candidates’ safety and efficacy. While *D. melanogaster* is a more challenging model, with lower throughput, it offers a complex scenario for studying drug susceptibility due to tissue homology, sex dimorphisms, and a conservative immune system. Taking all these features into account can help to evaluate the best timeline for your drug screening.

After these considerations, we will address all the aspects to take into account in the *D. melanogaster* model for compound screening in the infectious diseases field.

## 3 How to treat *Drosophila melanogaster*

**Figure 1:**
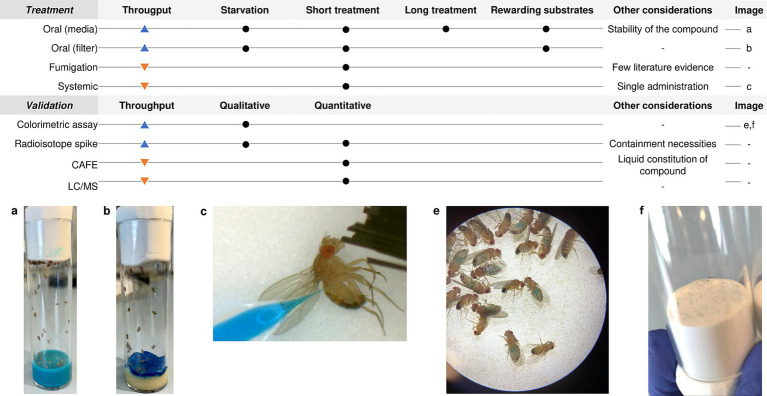
*Drosophila melanogaster* treatment and validation techniques.

Here we focus on the methodology under the treatment of infected flies, meaning the different treatment strategies that exist for *D. melanogaster* and some technical considerations such as which is the optimal drug concentration or which validation technique to use after treatment. Since the topic of this review is already very wide, we will not explain fly infection. If needed, you can consult an excellent review about the topic (Troha and Buchon, 2019). In Figure 1 you can find a summary of all techniques described in this part and in Tables 1, 2 all the information extracted from the articles discussed.

**Table 1:** Studies assessing infection treatment in *D. melanogaster*.

Infection	Compound	Action	Route	Treatment time	Concentration^1^	Measures	Host	References
Gram–positive								
*Staphylococcus aureus*	Methicilin; tetracycline	Standard	Oral	50 h	500–2000 μg/mL; 50–200 μg/mL	Survival	M/F	Needham et al. (2004)
*S. aureus*	Linezolid	Standard	Oral	8d	15–500 μg/mL	Survival	M/F	Diaz et al. (2013)
*S. aureus* (MRSA)	Linezolid	Standard	Oral	24 h pre.i. 7d	500 μg/mL	Survival	M/F	Ben-Ami et al. (2013)
*S. aureus*	Linezolid	Standard	Oral	18 h	500 μg/mL	Survival, pathogen load, Rapid Iterative Negative Geotaxis (RING), gene expression	M/F	Kaynar et al. (2016)
*S aureus; Candida albicans*	Plumbagin	Standard	Oral	24 h	40–80 μg/mL	Survival, pathogen load	F	Nair et al. (2016)
*S. aureus*	Nano Tigecycline/Chitosan–PRP Composite Hydrogel	Standard	Oral	24 h	–	Survival, pathogen load and fluorescence microscopy	F	Nimal et al. (2016)
*S. aureus* (MRSA)	Lantibiotic NAI–107	Standard	Systemic	3 h p.i.	100xMIC	Survival, pathogen load and gene expression	M	Thomsen et al. (2016)
*S. aureus*	Linezolid; dichloroacetic acid (DCA)	Standard	Oral	18 h; 7d	500 μg/mL	Survival and gene expression	M/F	Bakalov et al. (2020)
*S. aureus* (MRSA)	Capsosomes with vancomycin	Standard	Systemic	Coinjection	–	Survival	M/F	Tonkin et al. (2022)
*Bacillus cereus; B. anthracis; B. subtilis; Serratia liquefaciens; Escherichia coli*	Anthrax toxin component	Host	Oral	–	1 μg/mL	Survival	M/F	Alameh et al. (2020)
Gram–negative								
*Pseudomonas aeruginosa*	MPK1; MPK6 (Phages)	Standard	Oral	48 h	5×10^7^ PFU	Survival and pathogen load	M/F	Heo et al. (2009) and Jang et al. (2019)
*P. aeruginosa*	HWPB–1; HWPB–2; HWNPB–2; HWNPB–3; HWNPB–1; HWPB–3 (Phages)	Standard	Systemic	6 h p.i.	1.20×10^5^ PFU/fly	Survival	F	Lindberg et al. (2014)
*P. aeruginosa*	Baicalin	Virulence	Oral	14d	250 μg/mL	Survival	M/F	Zhang et al. (2021)
*P. aeruginosa*	Lactonase	Virulence	Oral	4d	400 μg/mL	Survival and pathogen load	M/F	Porzio et al. (2023)
*Salmonella Paratyphi* A	Fucoidan coated ciprofloxacin loaded chitosan nanoparticles (Fu–cCNPs)	Standard	Oral	48 h	4xMIC	Survival and pathogen load	M/F	Elbi et al. (2017)
Mycobacteria								
*Mycobacterium marinum*	Isoniazid; rifampicin; ethambutol; pyrazinamide; amikacin; dinitrobenzamide; ampicillin	Standard	Oral	15d	100–500 μg/mL	Survival, pathogen load and fluorescence microscopy	M/F	Oh et al. (2013a)
*M. abscessus*	Tigecycline; clarithromycin; linezolid; clofazimine; moxifloxacin; amikacin; cefoxitin; dinitrobenzamide; metronidazole	Standard	Oral	9d	100–500 μg/mL	Survival, pathogen load and fluorescence microscopy	F	Oh et al. (2014)
*M. marinum*	Ohmyungsamycins (OMS)	Host	Oral	–	1, 10 μM	Survival and pathogen load	M/F	Kim et al. (2017)
*M. marinum*	Diosmin	Standard	Oral	11d	250–2000 μg/mL	Survival, pathogen load, light microscopy	M/F	Pushkaran et al. (2019)
Fungi								
*Aspergillus fumigatus*	Voriconazol	Standard	Oral	24 h pre.i 10d	1,000 μg/mL	Survival, pathogen load, histopathological analysis	F (Toll^−^)	Lionakis et al. (2005) and Lionakis and Kontoyiannis (2010)
*Rhizopus oryzae*	Metformin	Host	Oral	8d	1291.64 μg/mL	Survival, body weight and light microscopy	F	Shirazi et al. (2014)
*Rhizopus oryzae*	Haemofungin	Standard	Oral	8d	14,400 μg/mL	Survival	M/F (Toll^−^)	Ben Yaakov et al. (2016)
*C. albicans*	Clioquinol	Standard	Oral	24 h pre.i. 7d	1,000 μg/mL	Survival and pathogen load	F (Toll^−^)	Pippi et al. (2019)
*C. auris*	Fluconazole; posaconazole	Standard	Oral	24 h pre.i. 7d	1,000 μg/mL	Survival, pathogen load and light microscopy	M/F (Toll^−^)	Wurster et al. (2019)
*Trichophyton rubrum; T. mentagrophytes; Microsporum canis; Nannizzia gypsea*	Terbinafine; itraconazole; clioquinol	Standard	Oral	24 h pre.i. 7d	1,000 μg/mL	Survival	F (Toll^−^)	da Costa et al. (2023)
Virus								
CPrV; DCV; VSV	Aspirin	Host	Oral	–	0.5–1 μg/mL	Survival and viral titers	M/F	Kong et al. (2023)
*West Nile Virus*	Insulin	Host	Oral	48 h pre.i. 10	57.33 μg/mL	Viral titers	M/F	Trammell et al. (2023)

1 The concentration values showed in this table, when possible, have been all expressed in μg/mL. We have used molecular weight values of the compound if required.

hours (h), days (d), pre-infection (pre.i.), post-infection (p.i.), Minimal Inhibitory Concentration (MIC), males (M), females (F).

**Table 2:** Main results from studies assessing infection treatment in *D. melanogaster* (Continuation of Table 1).

References	Main results
Gram-positive	
Needham et al. (2004)	Flies infected with *S. aureus* and treated with tetracycline or methicillin increased their survival in comparison with the non-treated flies. 2 mg/mL was the most efficient methicillin concentration. Lower tetracyline doses (200 μg/mL) were needed to obtain the same effect.
Diaz et al. (2013)	500 μg/mL of linezolid in fly food protected *D. melanogaster* against all *S. aureus* strains (either resistant and susceptible ones).
Ben-Ami et al. (2013)	Linezolid increased the survival rate of flies infected with MRSA independently of being Panton-Valentine leukocidin (PVL) positive or negative.
Kaynar et al. (2016)	*S. aureus* infected flies and treated with linezolid had better survival and lower bacterial burden 24 h after infection in comparison with the non-treated group. Nevertheless, in comparison with non-infected flies (sham) the geotaxis of the infected and treated ones were significantly lower only in the first 48 h. In addition, flies surviving sepsis showed persistent NF-kB activation and AMP expression, suggesting an effect on their significantly shorter lifespan in comparison with sham.
Nair et al. (2016)	Survival rate of both *S. aureus* and *C. albicans* infected *D. melanogaster* was increased until 80 and 75%, respectively, when infected flies were treated with plumbagin. Pathogen burden was reduced 1.5 and 2 logarithms (in a dose-dependent way) when flies were treated in comparison with the non-treated group although a bigger n is needed to achieve significance.
Nimal et al. (2016)	All survival, bacterial burden and fluorescence imaging showed significant reduction of the *S. aureus* infection affectation in those flies that were treated with the hydrogel of tigecycline-covered nanoparticles in comparison with non-treated ones. Nevertheless, those flies treated with the covered nanoparticles had an 80% survival probability and achieved an 80% reduction in bacterial count, in comparison with the flies only treated with tigecycline, which had a survival probability of over 90% and achieved a 100% reduction in bacterial count.
Thomsen et al. (2016)	A 100xMIC of the lantibiotic NAI-107 injected 3 h post-infection with MRSA improved fly survival curves in comparison with non-treated flies, and showed lower bacteria burden than vancomycin treatment 24 h post-infection.
Bakalov et al. (2020)	*D. melanogaster* lifespan after surviving sepsis from *S. aureus* infection (treated with linezolid) is prolonged after a 1-week exposure to dichloroacetic acid (DCA). DCA promotes a shift in antimicrobial peptides expression lead by a decrease in *drosomycin* and *cecropin A* expression and an increase in *defensin*.
Tonkin et al. (2022)	Capsosomes are seen to selectively deliver vancomycin to MRSA-infected flies and improve their survival rates by 50–90% in comparison with those infected and non-treated flies.
Alameh et al. (2020)	The anthrax toxin component PA_20_ confers better survival rates and resistance to infection by a wide variety of pathogens in *D. melanogaster* in comparison with non-treated flies, thus providing a broad-spectrum protection against infection.
Gram-negative	
Heo et al. (2009) and Jang et al. (2019)	Both MPK1 and MPK6 are protective against *P. aeruginosa* infection in *D. melanogaster* by inhibiting bacterial proliferation *in vivo* and enhancing the survival curves.
Lindberg et al. (2014)	*D. melanogaster* flies infected with *P. aeruginosa* and treated with each of the 6 environmental phages had significantly increased survival mean time (27.8–45.7 h) in comparison with the untreated group (22.8 h). In addition, authors found a positive correlation between the phage growth rate and its therapeutic efficacy. The faster the phage is able to grow *in vitro*, the better the phage is able to combat bacterial infection.
Zhang et al. (2021)	Survival rates of those flies infected with *P. aeruginosa* and treated with baicalin was significantly increased in comparison with the non-treated flies.
Porzio et al. (2023)	*P. aeruginosa,* when infecting *D. melanogaster* and in the presence of the enzyme lactonase, was significantly more virulent compared to those flies infected with *P. aeruginosa* and non-treated. Flies treated with lactonase had a significantly increased death rate. Nevertheless, *D. melanogaster* treated with enzyme showed a reduction in the number of *P. aeruginosa* recovered at the end of treatment. Being this reduction more evident when combined with tobramycin.
Elbi et al. (2017)	Fu-cCNPs treated *D. melanogater* previously infected with *Salmonella* showed a recovering in the survival curves in comparison with the non-treated group, and the maximum anti-bacterial activity without showing toxicity.
Mycobacteria	
Oh et al. (2013a)	Rifampicin, dinitrobenzamide, amikacin and isoniazid effectively extended the life span of *M. marinum* infected *D. melanogaster* in comparison with the non-treated ones. Those antibiotics showed to clear the fly from infection at day 11th of treatment.
Oh et al. (2014)	Tigecycline had the best *in vivo* activity against *M. abscessus* infection in *D. melanogaster*, followed by clarithromycin and linezolid, in a dose-dependent manner. They also showed reduction in CFU in the treated flies at day 6th of treatment.
Kim et al. (2017)	OMS-A improved survival of the flies when infected with *M. marinum* and reduced the bacterial burden of the infection in a dose-dependent manner.
Pushkaran et al. (2019)	The highest improvement of survival curves of infected with *M. marinum* and treated flies was given by the combination of diosmin with clavulanic acid in their highest doses (2 and 1 mg/mL respectively). By day 9 post-infection, infection was cleared in the treated flies.
Fungi	
Lionakis et al. (2005) and Lionakis and Kontoyiannis (2010)	The consumption by toll-deficient adult flies of voriconazol 24 h pre-infection and along 10 days post-infection decreased tissue fungal burdens detected by qPCR, histopahological analysis, and SEM and also showed better survival curves in contrast with the non-treated and infected with *A. fumigatus* flies.
Shirazi et al. (2014)	Administration of metformin to the diet of flies infected with *R. oryzae* led to weight loss, normalized glucose levels during infection, and was associated with decreased mortality and tissue fungal burden in comparison with non-treated *D. melanogaster*.
Ben Yaakov et al. (2016)	Results showed a significant difference in survival between flies infected with *R. oryzae* and fed with haemofungin-containing fly food versus those infected flies fed with regular fly food.
Pippi et al. (2019)	Clioquinol, successfully protected Toll-deficient *D. melanogaster* female flies infected with *C. albicans* when administered orally by significantly increasing the survival rates and reducing the fungal burden. Nevertheless, neurotoxicity must be further investigated.
Wurster et al. (2019)	Posaconazole is the drug that more effectively protected flies from *C. auris* infection in terms of survival and fungal burden.
da Costa et al. (2023)	The antifungal drugs (terbinafine, itraconazole and clioquinol) protected *D. melanogaster* from the infection with several fungal agents, except for *N. gypsea* whose survival curves did not differ from the untreated group.
Virus	
Kong et al. (2023)	Dietary supplementation with aspirin reduced viral proliferation in adult flies and showed longer survival curves in comparison with non-treated flies.
Trammell et al. (2023)	Oral treatment with insulin had an effect on reducing viral titers by West Nile Virus in wild-type *D. melanogaster* but not in virus-susceptible flies.

### 3.1 Oral treatment of adult flies

The most common and high-throughput method to treat the flies is to dissolve the drug either in the fly’s melted standard cornmeal or in a minimal medium (made with sucrose, agarose, and water) and let the flies feed *ad libitum* (Kruger and Denton, 2020). This methodology has the advantage of mimicking the most common route of drug administration in humans for treating local infectious diseases and also simplifies a lot the handling of the animal model. It is generally used for long-term exposure studies where flies are exposed to the drug food for more than 24 h (Diegelmann et al., 2017; Figure 1). When to administer the compound and the exposure time of the fly to the drug-complemented media will depend on the target infectious agent and its pathogenic cycle in the fly. In addition, it is well known that adding rewarding substrates, such as sucrose or yeast paste, to the drug-food preparation increases the possibility for a successful administration of the compound to the flies in addition to masking the possible bad taste of drugs (Pandey and Nichols, 2011). In this case, it is also important to consider the thermostability of the compound. If the compound is thermosensitive, then it should be added to the media surface when it is cold. This prolongs the media preparation process and requires considering the possible dilution of the drug into the media when calculating the necessary drug concentration (DeLoriea et al., 2023).

On the other hand, when the purpose of the study is to assess the effect of acute exposure to the drug, then the strategy is to limit the fly’s capacity to reach the nutrients so they only are fed with the compound of interest. Before being transferred to the treatment vials, adult *D. melanogaster* should be starved to ensure the predisposition of all the flies to eat the compound. This will favor the consumption of the fly of a large dose of the drug (Pandey and Nichols, 2011). The duration of the starvation as well as the duration of the exposure to the drug will also depend on the characteristics of your study. Some examples of this methodology are to cover the standard feeding media fully with a Whatman Filter Paper disk in which you put the compound solution in a proper concentration complemented with sucrose (Needham et al., 2004; Elbi et al., 2017), or let the vials medium-free and put a pile of folded papers or cotton balls previously soaked into a 2.5 mL solution containing the compound in a proper concentration (Troha and Buchon, 2019; Figure 1). These methods show variability in the amount of drug ingested and reduce screening throughput. Moreover, since these methods, together with the minimal media, do not allow the flies to receive all the nutritional intake they need, the exposure time and the duration of the study must be reduced to a safe time limit in order to not misinterpret efficacy results with malnutrition effects (Kruger and Denton, 2020).

#### 3.1.1 Methodologies to control *Drosophila melanogaster* food consumption

Although feeding techniques are widely employed for drug administration in Drosophila studies, there are several feeding-related issues. These include reduced feeding caused by flies favoring particular compounds, uncertainty about the amount of food ingested per fly, and ambiguity regarding the actual drug concentration reached in the tissues of the flies. In this regard, there are some techniques to address these potential issues.

Some authors performed a drug consumption control assay by using colored food vials. When eating, the dye is visible through the abdomen of the fly, and consumption can be monitored and photographed using a stereomicroscope in a qualitative way (DeLoriea et al., 2023; Figure 1). To make it semi-quantitative Shell et al., presented the Con-Ex method for determining the intake of colored solid media consumption in drosophila. They collected both the internal (consumed) dye in the flies by homogenization and the external (excreted) dye on the walls of the tubes and measured absorbances in a spectrophotometer (Shell et al., 2018). Alternatively, food could be also spiked with radioisotopes, this permits the measurement of the solid media consumption in flies based on the internal accumulation of a radioactive tracer through the fly cuticle with a scintillation counter. Radioisotope accumulates in the fly longer than colorimetric dyes; however, flies need special containment (Deshpande et al., 2014; DeLoriea et al., 2023).

Another extended methodology is the Capillary Feeder Assay (CAFE). It consists on the measurement of the food consumption by *D. melanogaster* by graded glass capillaries filled with liquid food placed on the top of the vial. Measurement of food consumption is based on the ratio between the capillary length and the difference on liquid distance in the capillary when the assay is finished, always taking into account the evaporative loss (Diegelmann et al., 2017). Some authors have used this technique in the study of treatment efficacy in *D. melanogaster* (Spindler et al., 2012; Van den Bergh, 2022). However, this technique requires more technical hours which, together with the limitation of the number of flies that can be studied per tube, reduces its throughput capacity. Nevertheless, some modifications have been made in the literature to surpass the problem of the population studied (Spindler et al., 2012).

Although these are the most used techniques additionally incorporated to study drug consumption by flies in treatment studies, there are many more. If more information is required you can consult the extended review published about the topic (Mahishi and Huetteroth, 2019). All in all, the food consumption techniques, although surpassing the food intake quantification problem, focus more on the study of fly’s behavior when feeding. Its use in treatment studies in drosophila can be limited as they need specific equipment and cannot be performed in a high-throughput manner. However, we should take them into account as they are useful to validate the treatment protocol and verify that the flies are being fed.

#### 3.1.2 Proper drug concentrations for infectious disease treatment in *Drosophila melanogaster*

What dose of the drug should we test in the screening is a topic of major concern. Few articles have tackled the topic of the drug-concentration scaling between species (Nair and Jacob, 2016; Del Valle-Moreno et al., 2023). Dose translation always needs careful consideration of body surface area, pharmacological, physiological, and anatomical factors, pharmacokinetic parameters, metabolic function, receptors, and life span. These parameters vary among species, as well as among individuals within the same species. In addition, it is well known that features such as sex or mating status affect the response of the fly against infection and treatment (Arch et al., 2023).

In addition to the uncertainty of the amount of food ingested per fly as described above, another challenge of oral treatment is to control the actual concentration of the drug inside each fly. Pandey and Nichols (2011) have pointed out that physiologically effective concentrations might vary from 0.01 to 100 mM in the feeding substrate but actual physiological concentrations inside the fly could be much lower. Liquid chromatography/mass spectrometry (LC/MS) presents itself as a powerful tool to verify the actual concentration of drug reaching each fly in the oral treatment protocol (Hammad et al., 2011; Oh et al., 2013a). Oh et al. determined that *M. marinum* infected flies with free access to a medium with 500 μg/mL of isoniazid for 10 days had an average concentration of isoniazid in the hemolymph of 4.86–5.15 μg/mL per fly. The authors showed how this concentration efficiently inhibited the growth of *M. marinum* in the treated flies (Oh et al., 2013a).

To optimize treatment while taking into account the main objective of your study as well as the laboratory possibilities, it is important to first validate your treatment technique. Dose–response experiments allow the establishment of an initial effective range for the drug to have an effect on *D. melanogaster*. To do that, you examine at least three different concentrations of a known effective drug at logarithmic dilutions in the food (p.e. 0.01, 0.1, 1.0 mM) and choose an appropriate concentration based on those results for the full screening (Pandey and Nichols, 2011).

In infectious diseases field, infections can be tackled with variety of compounds having different mechanisms of action against the microorganism. In literature, bactericidal compounds for testing in Drosophila normally have an effective dose response of 500 μg/mL in feeding media. This has been shown with rifampicin, dinitrobenzamide, amikacin, and isoniazid against *Mycobacterium marinum* (Oh et al., 2013a); linezolid against systemic infection of *Staphylococcus aureus* (Ben-Ami et al., 2013; Diaz et al., 2013; Bakalov et al., 2020); or tigecycline, clarithromycin, and linezolid against *Mycobacterium abscessus* (Oh et al., 2014). Lower doses showed less or no effect at the level of survival and clearance of pathogen load. In some cases, bacterial infections might be tackled with higher doses of compound when the mechanism of action is not fully elucidated. This happened to Pushkaran et al. (2019) when treating flies infected with *P. aeruginosa* with diosmin, a repurposed drug normally used in cardiovascular diseases. Interestingly, higher doses may also be needed to cure fungal infections in *D. melanogaster*. In this case, the effective antifungal dose required in the fly media must be at least 1 mg/mL to tackle infections such as *Aspergillus fumigatus*, *Candida albicans*, or *Candida auris* among others (see Table 1; Lionakis et al., 2005; Lionakis and Kontoyiannis, 2010; Pippi et al., 2019; Wurster et al., 2019). However, when talking about host-directed therapies, effective doses needed to improve host fitness are lower. This has been shown in *D. melanogaster* with host-directed therapies such as aspirin and insulin against viral infections (0.5–1 μg/mL and 10 μM, respectively) (Kong et al., 2023; Trammell et al., 2023), ohmyungsamycins as autophagy activators in the fly against *M. marinum* infection (1–10 μM) (Kim et al., 2017), or the Protective Antigen of the anthrax toxin that has been demonstrated to protect flies from a wide spectrum of pathogens with 1 μg/mL of compound in the food (Alameh et al., 2020). In addition, compounds that target virulence factors on the pathogen have also been studied alone or in combination with antibiotics. Some examples are lactonases that hydrolyze *P. aeruginosa* quorum-sensing molecules (400 μg/mL) (Porzio et al., 2023), or baicalin that had its target on the Type III secretion system of *P. aeruginosa* (250 μg/mL) (Zhang et al., 2021), with a broader range of effective concentrations. Another therapeutic strategy currently under the scope is phage therapy. *D. melanogaster* has been identified as a good model system to test the therapeutic efficacy of phages (Jang et al., 2019). The appropriate treatment dose for phage therapy varies depending on the route of administration (oral and systemic) and the infection inoculum. This has been demonstrated in a *P. aeruginosa* infection model of *D. melanogaster*, where 1.20×10^5^ PFU/fly systemically or 5×10^7^ PFU orally have shown to be effective therapeutic doses (Heo et al., 2009; Lindberg et al., 2014; Jang et al., 2019). So, as may have been observed, each type of compound seems to have a range of effective concentrations in the oral treatment of *D. melanogaster*. If you know the mechanism of action of the compound you are testing, it is easier to determine the appropriate dose range for the compound by consulting the literature. In Table 1 we dissected the articles found in the literature about the treatment of infectious diseases in *D. melanogaster* (Table 1).

### 3.2 Other treatment strategies

#### 3.2.1 Aerosol administration

Although the oral treatment of flies is widely used, there are other treatment strategies that might fit better your study. One such method is called the fumigatus methodology. Macêdo et al. used it to perform a toxicity assay in *D. melanogaster* for the compound O-Eugenol. It consists of flies placed in flasks with sucrose and water *ad libitum*, those flasks have a counter-lid of polyethylene terephthalate (PET) on the screw cap to which a filter paper was fixed at the inner side of the cap for application of the different doses of the compound of interest. There, the compound volatilizes from the top reaching the fly’s respiratory system while the flies feed and hydrate on sucrose solution at the bottom of the flasks (Macêdo et al., 2022). This technique allows the study of aerosol-based treatments for, for example, respiratory infections. Nevertheless, for now, its use is limited to some toxicity assays and the throughput of the technique is low for screening assays.

#### 3.2.2 Nano-injection administration

In addition, it exists the possibility of treating the flies systematically by employing a nano-injector (Nanoject II, Drummond) into the abdomen of anesthetized flies, just as the systemic infection (Troha and Buchon, 2019; Figure 1). It must be taken into account that flies physiology can be impaired with more than two punctions, so this technique is limited to single compound administration. Thomsen et al. (2016) successfully assessed the effect of the Lantibiotic NAI-107 against methicillin-resistant *S. aureus* (MRSA) infection by injecting the bacteria in the soft tissue surrounding the front legs of the fly, and the treatment 3 h post-infection in the lower thorax. A similar study was also conducted by Lindberg et al. (2014) to evaluate the efficacy of phage therapy against a *P. aeruginosa* infection in the fly. Contrarily, Tonkin et al. (2022) used coinjection of both the treatment and the MRSA to study the sensitivity and specificity of capsosomes to deliver vancomycin only in the presence of MRSA. The systemic treatment is a much more time-consuming technique, so its use is appropriate in certain studies with specific experimental characteristics and objectives.

## 4 How to monitor the treatment of infectious diseases in *Drosophila melanogaster*

Due to the versatility of *D. melanogaster* as a model for infections, we can monitor their treatment in different ways, each one not exclusive of the rest. Which readout is optimal will depend on the conditions and purposes of your study. The advantages and disadvantages of the different techniques are detailed below. In addition, information about literature examples of human infectious disease drug testing in *D. melanogaster* is detailed in each part. You can find detailed information on these in Tables 1, 2.

### 4.1 Survival

Survival monitoring is, by far, the most commonly used technique for treatment studies in *D. melanogaster* as it permits the highest throughput and reliably shows fly fitness along the experiment. It is used in the toxicological evaluation of drug candidates, dose–response experiments, and infectious disease treatment monitoring, among others. It consists of daily monitoring of live/dead flies along the duration of the experiment, which normally lasts until the infection progresses in *D. melanogaster* and kills the flies in the non-treated group; this could be hours for high replicative pathogens such as *S. aureus* (15–25 h) or days for slow-growing pathogens such as *M. marinum* (approximately 10–20 days) (Dionne et al., 2003; Needham et al., 2004). Nevertheless, it will depend on the characteristics of your study as the virulence of the pathogen, the infectious route (oral or systemic), and the infective dose. Every 2–3 days flies must be placed in new tubes of medium with or without compound because those get sticky and pasty, which can trap and kill flies, leading to a misinterpretation of survival results. In addition, there is a risk of flies not being able to reach the compound as it may be hindered by the eggs laid by females in the medium, impairing treatment efficacy (Troha and Buchon, 2019). Suitable treatment can improve survival curves by 50–90% (Zhang et al., 2021; Tonkin et al., 2022). However, this will depend also on the exposure time to the treatment (pre-infection, immediately post-infection, or some days after the infection), and the compound dose. Of note, treatment can contribute to infection clearance at the expense of flies’ survival. Kaynar et al., performed oral treatment with linezolid along 18 h of *S. aureus*-infected flies and measured survival and CFUs on several time points post-treatment. Flies did not show *S. aureus* CFUs in the treated group on day 20th after treatment. Nevertheless, the infection and the treatment impaired the overall survival rate in comparison with the non-infected group (Kaynar et al., 2016).

The outcome of the data generated are survival curves. They represent the probability for an individual to survive to a given time as proportions of the total of live flies. The outcome is a dichotomous event, corresponding to 1 for die or 0 for live. Those flies that die within the first 2 h after infection should be excluded from the analysis as their death can be attributed to the severe injury of the wound due to injection and not to the infection (Troha and Buchon, 2019). Analysis can be calculated using different equations. The Kaplan–Meier method is a non-parametric method that displays the probability of survival as a function of time, this means, the probability of the event occurring at time *t* (Lee, 2023). This method is appropriate when your study does not have additional variables (covariates), for example when studying treatment versus control groups (Kong et al., 2023; Porzio et al., 2023). On the other hand, the Cox proportional hazards model is a semi-parametric method that uses hazard functions to give a sense of the risk of the event occurring for an individual at time *t* (Lee, 2023). The Cox model takes into account the covariates that might have an effect on survival. In treatment experiments, this method is normally represented by comparing the ratio of hazards between the treatment and the control group (Ferreira et al., 2014; Van den Bergh, 2022).

### 4.2 Pathogen load

In *D. melanogaster*, and in the infections field in general, host fitness (survival) not always reflects infection status in the fly. It can happen that, when treated, flies may have the same or worse survival rates in comparison with the non-treated group but clear the infection. This happened when Porzio et al. gave lactonases in combination with Tobramycin to *P. aeruginosa*-infected flies. Flies infected with *P. aeruginosa* in the presence of the enzyme had a significantly increased rate of *D. melanogaster* deaths. However, the same group of flies showed a reduction in the number of *P. aeruginosa* recovered at the end of treatment. This happened because the lactonases reduce the biofilm formed by *P. aeruginosa* in infection, favoring a rapid spread of the infection through the fly. At the same time, when lactonases were administered together with tobramycin, this resulted in higher susceptibility of planktonic *P. aeruginosa* to the antibiotic treatment (Porzio et al., 2023). In the same scenario, the trade-off between immunity and lifespan in *D. melanogaster* has been extensively studied. The investment by the host in immunity impairs its fitness attributes such as longevity and fecundity (Ye et al., 2009; Arch et al., 2023). In treatment, this can happen when host-directed therapies are administered, where an activation in flies’ immune system can end their life but also infection in a phenomenon known as cytokines storm, also evidenced in humans (Van De Leemput and Han, 2021). Contrarily, it may also happen that the compound does not display any antimicrobial effect in the infection but acts as host-directed therapy that favors tolerance, meaning, increases the lifespan of the fly against elevated pathogen load (Vincent and Dionne, 2021). This is why, to better elucidate the effect of a drug against infection (clearance, or tolerance and resistance induction), it is worth it to add pathogen load data to your survival data.

Pathogen load is mostly measured by counting the number of Colony Forming Units per fly at specific time points in the study (for example, pre-, and post-treatment). This is a good method when monitoring treatment because it shows the live bacteria remaining in the fly. The experimental procedure is perfectly detailed in Troha and Buchon (2019). Briefly, individual infected or infected and treated flies must be harvested, rinsed in ethanol 70%, homogenized in PBS, serially diluted, and plated in agar plates of the appropriate media until, after incubation, CFUs can be counted (Troha and Buchon, 2019). The addition of proper antibiotics on the media must be taken into account in order to get rid of the fly microbiota and detect only the pathogen of interest. From this point, tolerance and resistance can be measured in order to better elucidate treatment effect. While resistance strategies will aim at killing the pathogen or inhibit its proliferation, tolerance strategies will reduce the negative effect on fitness caused by the infection, but will not have an impact on pathogen fitness (Arch et al., 2022). Arch et al., defined tolerance as the slope of the regression between survival and pathogen load, with the flattest slopes representing increased tolerance. Resistance was measured by the authors as the Y-intercept of the regression line between the pathogen load upon death and the inoculation dose, with a lower Y-intercept as a measure of more resistance (Arch et al., 2023).

It can happen that culture-dependent methods are not suitable for your study. For example, this can occur when you cannot distinguish between the microbiota of the fly and the pathogen of interest by specific culture plate or antibiotics. Alternatively, the pathogen may be distinguishable by plating but it may require long incubation times and favorable temperatures that allow microbiota to select for antibiotic-resistant clones and grow, making it difficult to count the microorganism of interest. In these situations, pathogen load can be measured by quantification of microbial genes in fly extracts by PCR. Nevertheless, in treatment studies DNA detection is not a reliable technique to monitor treatment response as DNA is a stable molecule that survives long after cells have died (Honeyborne et al., 2011). Contrarily, 16S ribosomal RNA has a shorter half-life that has demonstrated parallelism with treatment and cell viability (Sheridan et al., 1998; Gordillo et al., 2006; Honeyborne et al., 2011; Azam et al., 2022). Some authors have already demonstrated the ability of the 16S rRNA to monitor treatment by performing a real-time Reverse Transcriptase quantitative Polymerase Chain Reaction (RT-qPCR) in tuberculosis patients’ sputum samples. Results showed a correlation between the detection of the gene and the time to positivity in MGIT and a good treatment follow-up (Honeyborne et al., 2011; Azam et al., 2022). Nevertheless, this technology has not been used yet in *D. melanogaster* treatment studies.

Qualitative manners of evaluating pathogen load are also present, such as the use of fluorescently labeled bacteria and their visualization in a fluorescence dissecting microscope. However, this is a lower throughput assay likely to be more relevant for validation of leads or when a more detailed analysis of the fly is required, for example, location and specific organ information. Fluorescence microscopy has been used by some authors to verify treatment efficacy against infections such as *M. marinum*, *M. abscessus* and *S. aureus* in *D. melanogaster* adult flies (Oh et al., 2013a, 2014; Nimal et al., 2016). Thus, this technique is commonly used to confirm and validate the results you are obtaining in the dose–response experiments rather than be used routinely to monitor treatment.

### 4.3 Immune response gene expression

Extra information can be extracted from our treatment study in *D. melanogaster* if we assess the immune response in the host elucidated by treatment. This is an advantage of the fly over other model hosts since the expression of any gene in *D. melanogaster* can be studied at specific time points (Tzelepis et al., 2013). This can be achieved by comparing endogenous RNA transcript levels between infected *D. melanogaster* and infected and treated flies by RT-qPCR (Troha and Buchon, 2019). It is worth it to point out here that it is difficult to obtain results from individual flies. A pool of 3–10 flies will be necessary per sample and time point to reach sufficient RNA levels after the acid nucleic extraction (Troha and Buchon, 2019; Arch et al., 2023). The nucleic acid extraction can be done in multiple ways; you should check which one fits with your laboratory conditions and works well in your samples. The Trizol method is very efficient but also some commercial kits can provide high levels of purified RNA with less background noise, that can interact with PCR reagents (Livak and Schmittgen, 2001; Moskalev et al., 2015; Wu et al., 2016; Hemphill et al., 2018; Green and Sambrook, 2020; Arch et al., 2023). At this point, after a retrotranscription, a qPCR of the genes of interest can be done (Tajadini et al., 2014).

A myriad of immune-related genes of *D. melanogaster* can be studied. When elucidating treatment efficacy, it is of interest to study the change of expression of the typically expressed genes under infection in both flies that are infected and flies infected and treated. Genes such as *drosomycin (drs), metchnikowin* (*mtk*) and *defensin (def)* are known to increase their expression after a Gram-positive or fungal pathogen infects *D. melanogaster* by an upregulation of the Toll pathway (Lee and Edery, 2008; Hedengren-Olcott et al., 2004; Kaynar et al., 2016). Contrarily, when a Gram-negative pathogen infects the fly, AMPs such as *diptericin (dpt), attacin (att),* and *cecropin (cec)* are expressed by activation of the Imd pathway (Gottar et al., 2002; Mulcahy et al., 2011; Tapadia and Verma, 2012). In addition, the JAK/STAT pathway, involved in diverse biological processes, induces the transcription of Turandot stress genes, Upd3 (a cytokine analog of mammalian IL-6 and also related to cellular immunity), and some antiviral genes such as *vir-1* (Agaisse et al., 2003; Chakrabarti et al., 2016; Lopez et al., 2018). Regarding cellular immunity, the Imaginal morphogenesis protein late 2 (Impl2) has been recently related to the activation of phagocytes (Harsh et al., 2019; Krejčová et al., 2023), and Atg8a with autophagy (Tsapras et al., 2022; Demir and Kacew, 2023). Once qPCR is performed, the relative transcript levels of target genes are calculated using the 2^−ΔΔCT^ method with *rpl32* as the reference gene for the normalization of data (Livak and Schmittgen, 2001). A list of some relevant genes in the infectious field, with their amplification primer pairs for qPCR, is available in Table 3.

**Table 3:** List of primers for common genes studied when monitoring infections in *D. melanogaster*.

Gene	Primer forward (5′–3′)	Primer reverse (5′–3′)	Pathway	References
*rpl32*	ACAGGCCCAAGATCGTGAAG	TCGACAATCTCCTTGCGCTT	Ref. gene	Arch et al. (2023)
*drs*	CCAAGCTCCGTGAGAACCTT	CAGGTCTCGTTGTCCCAGAC	Toll pathway	Arch et al. (2023)
*mtk*	AACTTAATCTTGGAGCGA	CGGTCTTGGTTGGTTAG	Toll pathway	Troha and Buchon (2019)
*def*	TCTCGTGGCTATCGCTTTTGC	CCACATCGGAAACTGGCTGA	Toll pathway	Lee and Edery (2008)
*dpt*	GGCTTATCCGATGCCCGACG	TCTGTAGGTGTAGGTGCTTCC	Imd pathway	Arch et al. (2023)
*attA*	CCCGGAGTGAAGGATG	GTTGCTGTGCGTCAAG	Imd pathway	Troha and Buchon (2019)
*CecA1*	GAACTTCTACAACATCTTCGT	TCCCAGTCCCTGGATT	Imd pathway	Troha and Buchon (2019)
*totA*	CCCAGTTTGACCCCTGAG	GCCCTTCACACCTGGAGA	JAK/STAT	Troha and Buchon (2019)
*upd3*	GCAAGAAACGCCAAAGGA	CTTGTCCGCATTGGTGGT	JAK/STAT	Arch et al. (2023)
*impl2*	GCCGATACCTTCGTGTATCC	TTTCCGTCGTCAATCCAATAG	JAK/STAT	Arch et al. (2023)
*vir-1*	GATCCCAATTTTCCCATCAA	GATTACAGCTGGGTGCACAA	JAK/STAT	Troha and Buchon (2019)

To emphasize the importance of gene expression analysis in treatment studies, a few examples are detailed here. To Bakalov et al., the measurement of different immune genes served to elucidate which were the mechanisms underlining the increase in survival curves experienced by *S. aureus*-infected flies treated with linezolid and DCA for 1 week in comparison with flies only treated with linezolid. The authors showed that flies on a DCA diet experienced a significant decrease in expression of *drosomycin* and *cecropin A* and an increase in *defensin* in comparison with those infected flies growing on a regular diet (Bakalov et al., 2020). Along the same line, it has been demonstrated that infected flies treated with linezolid showed a focused and maintained increment in expression in comparison to sham, which corresponded with the shorter lifespan of these groups (Kaynar et al., 2016). Similarly, Thomsen et al. (2016) showed that the treatment efficacy of lantibiotics nisin and NAI-107 was correlated with a decrease in expression levels of the immune response genes *drosomycin*, *cecropin A*, and *attacin B* in flies infected with *S. aureus*. If the compound is a HDT the gene expression data brings a lot of information on how is the host actually reacting to the treatment, which, in this case, is expected to have a direct effect on the progress of the infection. When *D. melanogaster* is previously challenged with heat-killed *E. coli*, then flies showed protection against the consequent infection by both *Enterococcus faecalis* and *P. aeruginosa* mainly moved by a synergistic action of Toll and Imd pathways (Wen et al., 2019). Altogether, the gene expression gave an additive informative value to what was happening in the host during and after treatment, which better helped to elucidate pathogenic phenotype.

## 5 Cost-effectiveness of a treatment study in *Drosophila melanogaster*

In this review, we have described all possibilities for a treatment efficacy study against infections in *D. melanogaster*. Here, we will elaborate on how different protocol decisions affect the cost-effectiveness of the study and define the considerations needed for choosing the optimal method depending on the characteristics of the study. We have defined cost-effectiveness as a combination of the throughput capacity, the cost, and the information taken from the study.

When analyzing the impact of treatment against a rapid-growing microorganism, which infection will progress rapidly in *D. melanogaster*, a short treatment (up to 48 h) is enough to see an effect with both survival and CFUs (Kaynar et al., 2016; Elbi et al., 2017). So, a shorter time is necessary to analyze one compound. This increases the throughput and decreases the cost of the study. Differently, when the infection is done with a non-virulent or a slow replicative pathogen, the pathogenic cycle is longer, which increases survival monitoring time (from 24 h to 15 days) and treatment is also extended, so you need a higher amount of compound (reminding that fly media tubes are changed every 2 days) (Oh et al., 2013a, 2014; Pippi et al., 2019).

Another cost factor is the necessary dose of the compound to treat the infection. The calculations of how much drug quantity you need to obtain a certain concentration in fly media are perfectly explained in Kruger and Denton (2020). There you can see that to do a solution of 1 mM you will need almost 50 mg of compound inside the media to obtain 10 tubes (Kaynar et al., 2016; Kruger and Denton, 2020). This demonstrates that compounds requiring higher doses, for example, those needed to assess antibiotics or antifungals as discussed earlier, increase the cost of the study directly related to the production cost of the compound. In addition, the administration route of the treatment can also affect the costs of the study. Oral treatment of flies through a filter imbibed in the treatment solution will waste less compound, but this can only be used with short studies (fast pathogens), as the filter prevents the fly from eating the standard nutritive media. When the study lasts days, the compound mixed within the standard cornmeal of flies is the best option. However, it slightly compromises the cost of the study as you are using more compound than the flies will actually ingest, leaving dead volume of compound in the medium that will be thrown away.

Paying attention to the techniques used for the screening of compounds against infections in *D. melanogaster*, survival is the one permitting the highest throughput. It allows, in the best-case scenario, to screen thousands of drugs per week (Pandey and Nichols, 2011). However, as discussed earlier, by only assessing survival you might lose information about the infection progress. Lower throughput assays, such as pathogen load measurement, become crucial if you are looking for quality final compounds in your screening. Thus, the addition of pathogen load to the outcome of the study is a cost-efficient movement, as it will increase the procedural time but it will provide valuable information. Pathogen load is mostly measured pre-and post-treatment; however, it is confusing to talk about clearance of the infection, a third time point post-treatment might be useful to elucidate the long-term effects of this treatment (Kaynar et al., 2016; Bakalov et al., 2020). Gene expression can be reserved for the study of the effect of some compounds of interest on fly immunity when the main objective is to elucidate the mechanism of action, as it increases both the throughput (with handling time) and the cost due to the high cost of reagents. If any validation of your protocol is done, the colorimetric analysis combines the highest throughput and lowest cost (DeLoriea et al., 2023). However, if you are experiencing some incongruencies in the results it must be useful to check by LC–MS the exact quantity of compound achieving fly tissues.

As the last variable to take into account, studies can be done in one or both *D. melanogaster* sexes. Although the latter doubles the resources needed, it might be necessary as sexual dimorphism is a fact not only in *D. melanogaster* but also in other species such as mice and humans (Vázquez-Martínez et al., 2018). In *D. melanogaster*, like in mammals, X and Y sex chromosomes influence immune responses, with many genes encoding innate signaling proteins located on chromosome X and showing sex-specific induction following fungal or bacterial infections (Klein and Flanagan, 2016; Belmonte et al., 2020). Generally, human adult females mount stronger innate and adaptive immune responses than males, which results in faster clearance of pathogens and greater vaccine efficacy in females (Klein and Flanagan, 2016). Nevertheless, sex bias in human infectious diseases has been demonstrated according to the type of pathogen and the type of response mounted by the host, as happens in *D. melanogaster* (Taylor and Kimbrell, 2007; Duneau et al., 2017). In addition, in both species, humans and *D. melanogaster*, sex differences have also been attributed to the action of steroid hormones and the trade-off between hormonal regulation and immunity has been extensively reported (Regan et al., 2013; Klein and Flanagan, 2016; Arch et al., 2023). In *D. melanogaster* model, *Garlapow* et al., demonstrated that there is sexual dimorphism in food intake, with females generally consuming more than males (Garlapow et al., 2015). At the same time, males require less nutrients for its maintenance and lifespan (Wu et al., 2020). Thus, traits such as size, immune response to a challenge, or food intake are different between *D. melanogaster* sexes and can affect the overall treatment result (Garlapow et al., 2015; Belmonte et al., 2020). Studying the treatment effect of a certain compound on only one sex assumes that the same results will occur in the other sex. However, factors such as effective concentration, response to the drug, or other treatment necessities may not be the same in *D. melanogaster*, nor on its scalation to mammals.

**Figure 2:**
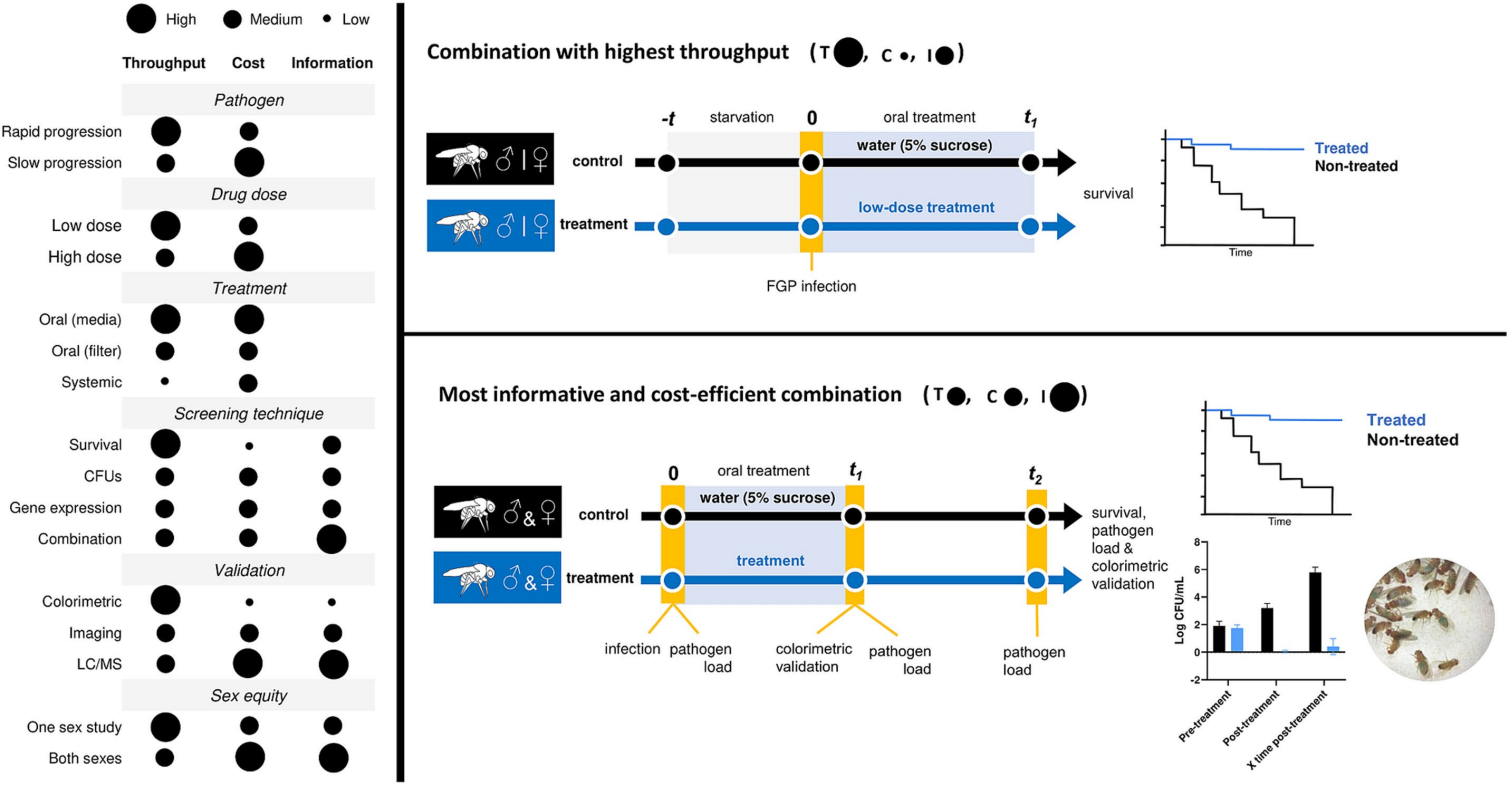
Variables that condition the experimental outcome of drug screening studies in *D. melanogaster*. Throughput (T), cost (C), information (I).

With all of the above, fit the study necessities to all the given possible options is crucial to build up a suitable treatment protocol. If you are aiming a high-throughput screening, mimicking mammal cell culture *in vitro* assays, the best option is to infect *D. melanogaster* with a fast-growing pathogen, previously starved for 2–18 h, perform an oral treatment and monitor survival along the course of the infection. Contrarily, if you prioritize obtaining quality compounds from the screening the most informative and cost-efficient combination could be infection with the pathogen, followed by a pathogen load measurement-previous to treatment-and an oral treatment with the compound. Then pathogen load is measured again at the end of the treatment and survival is monitored along the course of infection. In Figure 2 you can find how each variable can affect cost-effectiveness in treatment studies and the two combinations proposed (Figure 2). When comparing the outcome of treatment efficacy experiments in mammal models—such as survival rates, clinical symptoms, body weight loss, pathogen load, and tissue damage—to our proposed experimental options with *D. melanogaster*, it becomes evident that our approach enhances drug screening quality while reducing the need for expensive mammal experiments (Bui et al., 2024). This ultimately cuts down the overall cost of drug development without sacrificing the level of information obtained. Additionally, it allows for the assessment of more compounds per experiment.

## 6 Technical considerations and limitations of the model

Throughout the review, in addition to justifying all the advantages of *D. melanogaster* in the context of drug screening performance, we have also addressed the various limitations of the model. On one hand, there are some limitations associated with fly physiology. For example, the inability of flies to mount an adaptive immune response, or their structural and anatomical complexity compared to other animal models. The latter conferring both a disadvantage when talking about throughput, or an advantage if we refer to what this complexity can offer to the treatment efficacy outcome. On the other hand, there are some limitations strongly linked to the treatment methodology of the flies. Many issues arise due to the oral nature of this treatment. This includes the uncertainty of the amount of compound reaching each fly and the inefficiency of the current oral delivery format, which results in a significant waste of the compound. To provide a comprehensive understanding of the challenges and considerations when using the model, we have compiled the limitations into Table 4, along with comment–proposed solutions that the model offers to them. This table includes the section where each limitation is further elaborated.

**Table 4:** List of technical considerations and limitations of the *D. melanogaster* model for drug screening studies.

Limitation	Comments – proposed solutions	Section
Fly physiology		
Absence of adaptive immune response	Leading model in the study of innate immune responses conserved with mammals.	2
Lower throughput compared to other models	(a) The *D. melanogaster* model has a well-conserved innate immune response and anatomical tissues, providing valuable advantages for evaluating drug efficacies compared to other models. (b) The throughput of each *D. melanogaster* experiment is dependent on their outcomes: survival > survival and CFUs > survival, CFUs, and gene expression.	2.1; 5
Sexual dimorphism influences outcomes	(a) The sexual dimorphism of *Drosophila melanogaster* brings the model physiologically closer to that of higher organisms. (b) Testing the efficacy of the drug in both sexes allows you to customize treatment features such as dosage or treatment time for each sex’s necessities, but reduces the throughput.	5
Limited drug concentration scalability	*D. melanogaster* provides valuable insights into drug efficacy, PK/PD, and intestinal permeability. However, dose escalation should be done in models with better individual dosage control.	2
Treatment methodology		
Dependence on fly feeding behavior	Some rewarding substrates (yeast or sucrose) should be added to the media to increase attraction possibilities.	3.1
Interference between compounds and *D. melanogaster* growth conditions.	(a) Thermosensitive compounds may not be added to the melted media to prevent denaturation. (b) Photosensitive compounds may degrade during the experiment. Shorter experiments (acute infection) are recommended for testing those. (c) Using more than 0.1% of DMSO can be toxic to flies, so careful dosing is required when dissolving and preserving drugs.	3.1
Uncertainty in individual compound dosage/concentration	(a) Conduct a proper dose–response experiment to determine the optimal dose and time of exposure for each specific protocol. (b) Colorimetric assays are an effective qualitative method to confirm compound ingestion. (c) Mass spectrometry can be used to validate compound absorption and distribution in each fly, providing PK/PD information. (d) The Capillary Feeder Assay (CAFE) offers information on the feeding pace of each group of flies, allowing for a more controlled environment.	3.1.1; 3.1.2
Excess compound usage	Mix the compound into a yeast paste (rewarding substrate) and apply it to the surface of the media. This reduces compound use while maintaining the concentration.	5

## 7 Discussion

This review is an overview of the current methods for study treatment against infections in *D. melanogaster*. We have discussed the main advantages and disadvantages of the model in this regard. The fruit fly shares a high degree of genetic conservation with mammals and is a host of both natural and human pathogens, making it a promising tool for studying host-pathogen interactions and the timeline of new drug discovery. Its abundance of genetic tools, low-cost maintenance, and the throughput permitted in screening studies also contribute to its potential in this area (Buchon et al., 2014; Troha and Buchon, 2019). *D. melanogaster* not only offers to fill the gap between *in vitro* and mammalian models but works as an excellent selection point for high-quality compounds as it selects for compounds with already good oral availability and intestinal permeability, metabolic stability, good pharmacodynamic and pharmacokinetic values, and low toxicity (Pandey and Nichols, 2011; Tzelepis et al., 2013). All types of compounds, including host-directed therapies, antibiotics, and antivirulence agents, have been shown to impact the progression of infection due to enhanced innate immune response, decreased microbial virulence, or bactericidal activity when tested in *D. melanogaster* (Table 1). All of this together opens drastically the possibilities of the model allowing the study of many new compounds or even repurposed drugs against infections (Pushkaran et al., 2019).

Among all the available methodologies to treat flies, we have seen that the oral treatment methodology is the one with the best performance results. It is rapid, reproducible, and permits the highest throughput in comparison with other also used methodologies such as systemic treatment or the quantifiable oral treatment by Capillary Feeder Assay (CAFE) (Diegelmann et al., 2017). However, this is not the only aspect to take into account when deciding which is the best treatment protocol. Variables such as the pathogen virulence, the compound molecular concentration, or what to assess in the model must be considered to reach the optimal drug testing strategy according to your specific study conditions. While single survival monitoring of infected versus infected and treated flies is the most efficient approach to achieve the highest throughput in your study, it is important to incorporate specific time points for measuring pathogen load during the experiment in order to obtain more comprehensive information from your protocol in a cost-efficient manner (Figure 2). Moreover, sexual dimorphism is a universal phenomenon across all species. Therefore, when using the *D. melanogaster* model for drug screening, it is important to conduct tests on both male and female subjects because the effective concentrations and treatment needs may vary between the two sexes (Garlapow et al., 2015; Belmonte et al., 2020).

Nevertheless, some general limitations should be considered and further studied if we want *D. melanogaster* to be the model of preference for these kinds of studies. It is difficult to measure the amount of compound reaching each fly, and this number is dependent on the physiological variability of each individual. There is a need for new methodologies that reduce the amount of compound used per experiment, as 50 mg compound per experiment will not always be possible nor cost-effective, and with this methodology, you are using more compound than the flies will ingest (Kruger and Denton, 2020). A collaborative effort is essential to overcome these obstacles and establish the fly model as a benchmark for screening treatments against infections.

## Footnotes

^1^https://nc3rs.org.uk/who-we-are/3rs
